# Study in the iron uptake mechanism of *Pasteurella multocida*

**DOI:** 10.1186/s13567-025-01469-0

**Published:** 2025-02-13

**Authors:** Xiangxiang Shen, Lijun Guan, Junfeng Zhang, Yun Xue, Lifang Si, Zhanqin Zhao

**Affiliations:** 1 Key Lab of Animal Bacterial Infectious Disease Prevention and Control Technology, College of Animal Science and Technology, Henan University of Science and Technology, Luoyang, 471003 China; 2 Key-Disciplines Lab of Safety of Environment and Animal Product, College of Animal Science and Technology, Henan University of Science and Technology, Luoyang, 471003 China

**Keywords:** *Pasteurella multocida*, iron uptake mechanism, siderophore, ExbB–ExbD–TonB, Fur

## Abstract

*Pasteurella multocida* infects a wide range of animals, causing hemorrhagic septicemia or infectious pneumonia. Iron is an essential nutrient for growth, colonization, and proliferation of *P. multocida* during infection of the host, and competition for iron ions in the host is a critical link in the pathogenesis of this pathogen. In recent years, there has been significant progress in the study of the iron uptake system of *P. multocida*, including its occurrence and regulatory mechanisms. In order to provide a systematic theoretical basis for the study of the molecular pathogenesis of the *P. multocida* iron uptake system, and generate new ideas for the investigation and development of molecular-targeted drugs and subunit vaccines against *P. multocida*, the mechanisms of iron uptake by transferrin receptors, heme receptors, and siderophores, and the mechanism of expression and regulation of the *P. multocida* iron uptake system are all described.

## Introduction

*Pasteurella multocida* is a short, rod-shaped, Gram-negative, conditionally pathogenic bacterium [1]. Based on the capsule and lipopolysaccharide (LPS) antigen, *P. multocida* is divided into five capsular serogroups (A, B, D, E, and F) and sixteen LPS serovars (1–16) [2, 3]. *P. multocida* is capable of infecting a wide spectrum of domestic animals (cattle, sheep, poultry, pigs, rabbits, dogs, and cats) as well as humans, causing pasteurellosis, which is characterized by infectious pneumonia and hemorrhagic septicemia, resulting in enormous economic losses to the farming industry [4]. Iron ions are essential elemental factors for the colonization and proliferation of *P. multocida* during host infection. The iron uptake system of *P. multocida* is important to the mechanism of infection and immunity that characterizes the host–pathogen interaction, and has received extensive attention from researchers [5–7]. Iron participates in DNA and protein biosynthesis, biofilm formation, redox and electron transfer activities, and is required for growth and metabolism in microorganisms and animals [8, 9]. In the interaction between *P. multocida* and its host, iron has a crucial role in promoting bacterial growth, reproduction, adhesion, and expression of virulence factors [10–12]. The ability of bacteria to acquire iron is an important factor in their pathogenicity [13]. However, due to the host’s nutritional immunity, *P. multocida* frequently experiences iron deficiency during host infection. For this reason, bacteria have evolved multiple absorption mechanisms to collect iron ions from the host's transferrin (Tf) and hemoglobin (Hb) [14, 15]. In this paper, we review the development of three iron ion acquisition pathways, iron-uptake-related protein activities, and regulatory variables in *P. multocida.* From the perspective of interfering with bacterial iron uptake, a systematic study of the iron uptake mechanism of *P. multocida* will help generate new ideas for the development of innovative antimicrobial medicines and biologics.

## Biological functions of iron in bacteria

Iron is one of the most prevalent metallic elements on Earth, and it is typically present in either an oxidized (Fe^3+^) or reduced (Fe^2+^) state, which makes iron ions widely used in biological systems. Iron ions, are typically attached to proteins or as part of iron–sulfur clusters or heme groups, and are involved in a variety of important biological processes in microorganisms, including aerobic respiration, ATP synthesis, electron transfer, DNA replication, and protein synthesis [16]. Thus, iron is a necessary factor for bacteria to perform their own metabolism and functions.

### Functions of iron–sulfur clusters in bacteria

Iron–sulfur clusters are among the oldest structures found in all living organisms, and they are protein–metal clusters with crucial regulatory or catalytic functions. Functional investigations of iron–sulfur clusters using *P. multocida* as a model are rare, although clusters in other bacteria are better understood. Common structures in bacteria include [4Fe-4S], [3Fe-4S], and [2Fe-2S], which can be substituted, and hence, electron transferred [17]. Iron–sulfur clusters can stabilize some functions of bacterial proteins; for example, [4Fe-4S] clusters provide a more stable DNA-binding site for *Escherichia coli* nucleic acid endonuclease III [18]. Iron–sulfur clusters also shield proteins against proteolytic degradation by proteases. For example, [4Fe-4S] clusters provide aminotransferases in *Bacillus subtilis* with stable structure and activity, while disruption of clusters causes aminotransferase destruction [19]. Iron–sulfur clusters play a role in gene expression and regulation. For example, the rhizobial iron regulator A (RirA) requires an intact [3Fe-4S] cluster to regulate DNA transcription [20]. Superoxide response protein in *E. coli* functions as a signaling factor and transcriptional activator, and its function and activity are dependent on the [2Fe-2S] cluster [21]. *P. multocidia* contains homologues of these proteins, and the annotation information for these homologs is accessed in GenBank (endonuclease III, accession number AIN49160.1; aminotransferase accession number VEE36784.1; SoxR, accession number XHR73116.1).

### Functions of heme in bacteria

Heme is a porphyrin complex containing ferrous ions, which is found in animals, plants, and microorganisms, and sufficient levels of heme are favorable for promoting the growth of *P. multocida* [22, 23]. Heme functions as a cofactor in the biosynthesis of enzymes such as bacterial oxidase, peroxidase, and the level of heme regulates enzyme activity [24]. Oxidases are involved in bacterial aerobic respiration, while peroxidase genes are involved in bacterial metabolism, biofilm formation, and bacterial motility, as well as in host colonization and infection [25, 26]. 5-Hydroxytryptophan is the chemical precursor of many bioactive substances in bacteria, and heme can use the redox capacity of iron ions to hydroxylate the indole C5 position of tryptophan to form 5-hydroxytryptophan in the presence of O_2_ or H_2_O_2_ [27]. The enzymes indicated above are also found in *P. multocida* [28]*,* this demonstrates how vital iron is for this bacterium.

### Effects of iron shortage on *P. multocida*

The ideal concentration of Fe^2+^ for bacterial growth is around 10^–6^ mol L^−1^; below which, the phenomenon known as iron starvation occurs [29]. The solubility of Fe^2+^ at pH 7 is 1.4 × 10^–9^ mol L^−1^, and the concentration of Fe^2+^ in the host is significantly lower, which does not meet the bacterial requirement [30]. Iron is involved in the proliferation, adhesion, and virulence of *P. multocida* in the host. Under iron-limited conditions, *P. multocida* growth is inhibited, the thickness of the bacterial capsule is clearly reduced, and virulence is significantly decreased. In contrast, LPS synthesis increases considerably in low-iron conditions. LPS is an essential factor for the adhesion of bacteria in Pasteurellaceae to their hosts, and a sequence of modifications in *P. multocida* under iron-limiting conditions make it easier for it to adhere to its host [10, 11]. Outer membrane protein components of *P. multocida* are affected by iron, and under iron-restricted conditions, they express several iron-regulated outer membrane proteins (e.g., transferrin receptor and heme receptor) to capture iron ions from the host and defend against the negative effects of iron deficiency [31].

## Mechanism of iron uptake by the Tf receptor of *P. multocida*

**Figure 1:**
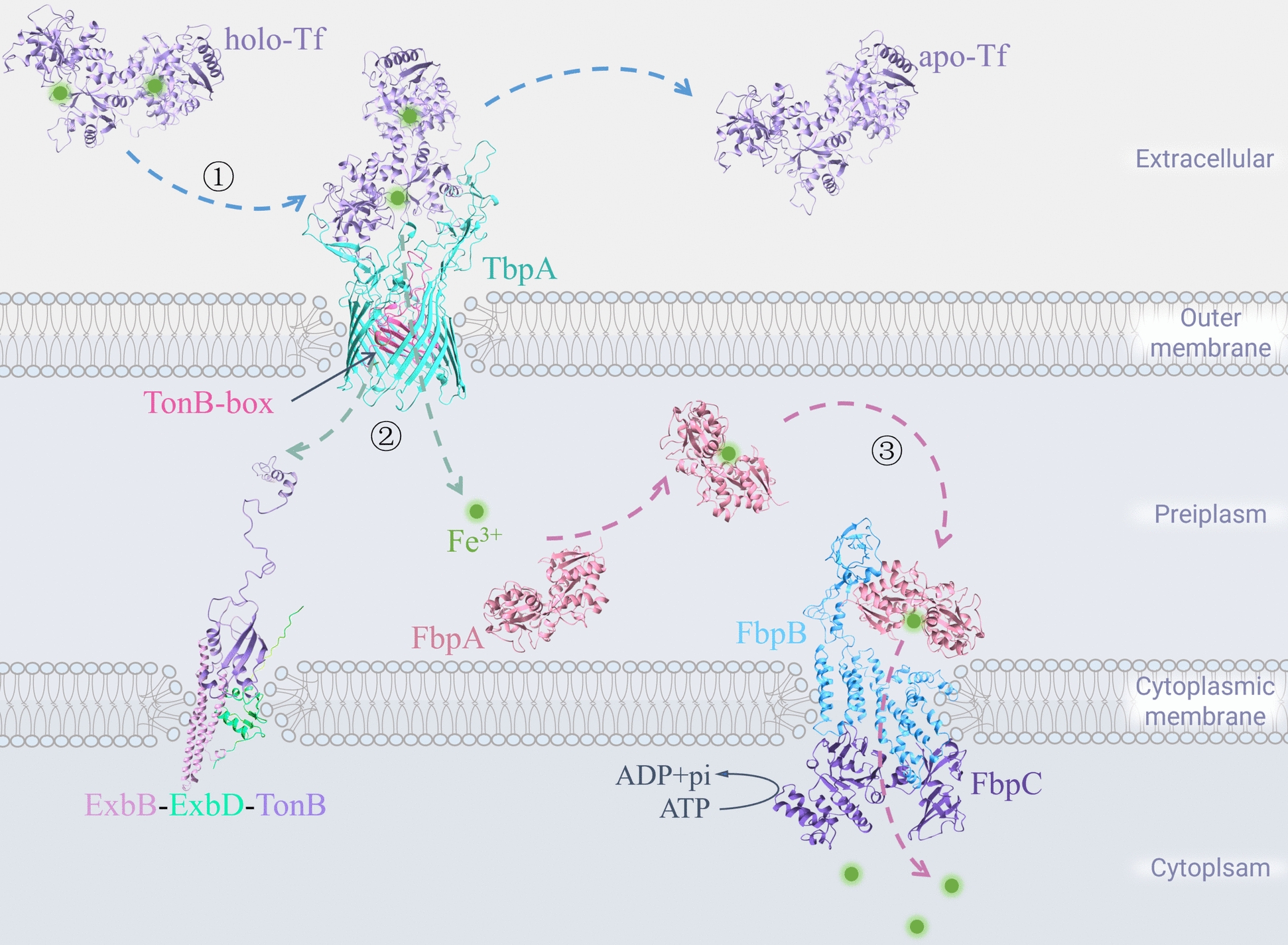
**Mechanism of iron uptake by the transferrin receptor in** ***P. multocida*.**

Tf-binding protein A(TbpA), a Tf receptor, is present on the outer membrane of *P. multocida* and can absorb Fe^3+^ from the iron-containing Tf (holo-Tf) of the host. Due to the lack of energy in the outer membrane of bacteria, iron ions must enter the periplasm via TbpA, where they are powered by the ExbB–ExbD–TonB system, and then enter the cytoplasm via the ATP-binding cassette (ABC) transporter for *P. multocida* to carry out its activities, such as growth, reproduction, and metabolism. Uptake of iron ions holo-Tf by *P. multocida* from host seems to require three steps: formation of a complex of holo-Tf with TbpA, entry of Fe^3+^ into the periplasm, and entry of Fe^3+^ into the cytoplasm via the ABC transporter system (Figure 1).

### Formation of complex of holo-Tf with TbpA

In the absence of free iron, *P. multocida* uses TbpA in its external membrane to absorb iron ions bound to the host holo-Tf. Tf is a glycoprotein synthesized mainly by host hepatocytes that can bind reversibly to Fe^3+^ [32]. TbpA is a TonB-dependent transmembrane receptor protein found in *P. multocida* [33]. It has a barrel-shaped structural domain (β-barrel) with 22 inversely parallel β-strands at the C terminus and an embedded plug at the N terminus [34]. The C-terminal β-barrel structural domain of TbpA specifically recognizes host holo-Tf and forms the transmembrane holo-Tf–TbpA complex [35, 36]. TonB box, the conserved region at the N-terminal plug-like structural domain of TbpA, undergoes conformational change, extends into the periplasm, and binds to TonB proteins on the cytoplasmic membrane to obtain energy, promoting Fe^3+^ transfer from the holo-Tf–TbpA complex to the periplasm [37, 38].

### Entry of Fe^3+^ into the periplasm

The TonB protein is anchored to the cytoplasmic membrane of *P. multocida* through an N-terminal hydrophobic α-helical structure, which binds to ExbB–ExbD proteins to form the ExbB–ExbD–TonB complex [39]. The complex provides energy for bacteria to exchange substances in the outer membrane and translocates nutrients such as iron ions across the membrane [34]. TonB binds to ExbB–ExbD via its N-terminal α-helical structure, and the transmembrane structural domains of ExbB–ExbD form a proton channel that transmits the proton motive potential energy from the cytoplasmic membrane to TonB. ExbB–ExbD on the cytoplasmic membrane, through rotational movement, induces the whiplash-like region of TonB (including the stretchable spacer region and the C-terminal domain) to oscillate in the periplasm and bind to the TonB-box region of the N-terminal end of the TbpA exposed in the periplasm to transfer energy to the TbpA. This results in opening of its plug and barrel structural domains, and opening and generation of a conduit in the barrel domain, allowing Fe^3+^ to enter the periplasm [34, 40, 41].

### Entry of Fe^3+^ into the cytoplasm via the ABC transporter system

The ABC transporter system can utilize the energy of ATP hydrolysis to transport substrates such as iron ions into the cytoplasm against the concentration gradient. Fe^3+^ is captured by *P. multocida* through TbpA, which enters the periplasm of the bacterium and is then transported into the cytoplasm via the FbpABC (An ABC transporter system). During this process, ferric binding protein A (FbpA) is free in the periplasm as a monomer under natural conditions and captures Fe^3+^ in the periplasm [42, 43]. FbpB is a hydrophobic transmembrane protein on the cytoplasmic membrane that is able to receive FbpA-delivered Fe^3+^. FbpC is a hydrophilic ATP-binding protein located on the inner side of the bacterial cytoplasmic membrane, which binds to FbpB to form a dimer and provides FbpB with the energy required for the transport of Fe^3+^ [44]. After entering the cytoplasm through the FbpABC, Fe^3+^ is reduced to Fe^2+^ for metabolism [45].

## Mechanism of iron uptake by the heme receptor of *P. multocida*

Most of the iron in animals is stored in heme [46]. Heme can bind globin to form hemopexins, such as Hb, myoglobin, and cytochromes [47, 48]. Hb is the primary source of heme absorption for bacteria [49]. *P. multocida* absorbs heme from host hemopexins via the heme transport system, transports it to the cytoplasm for catabolism, and obtains Fe^2+^ from it [7]. Heme transport is typically divided into two modes: direct transport, in which the bacteria directly take up host heme using heme receptors on the outer membrane; and indirect transport, in which the bacteria secrete high-affinity hemophores to the extracellular area, take up host heme, and deliver it to the heme receptors on the outer membrane.

### Direct heme transport system

**Figure 2:**
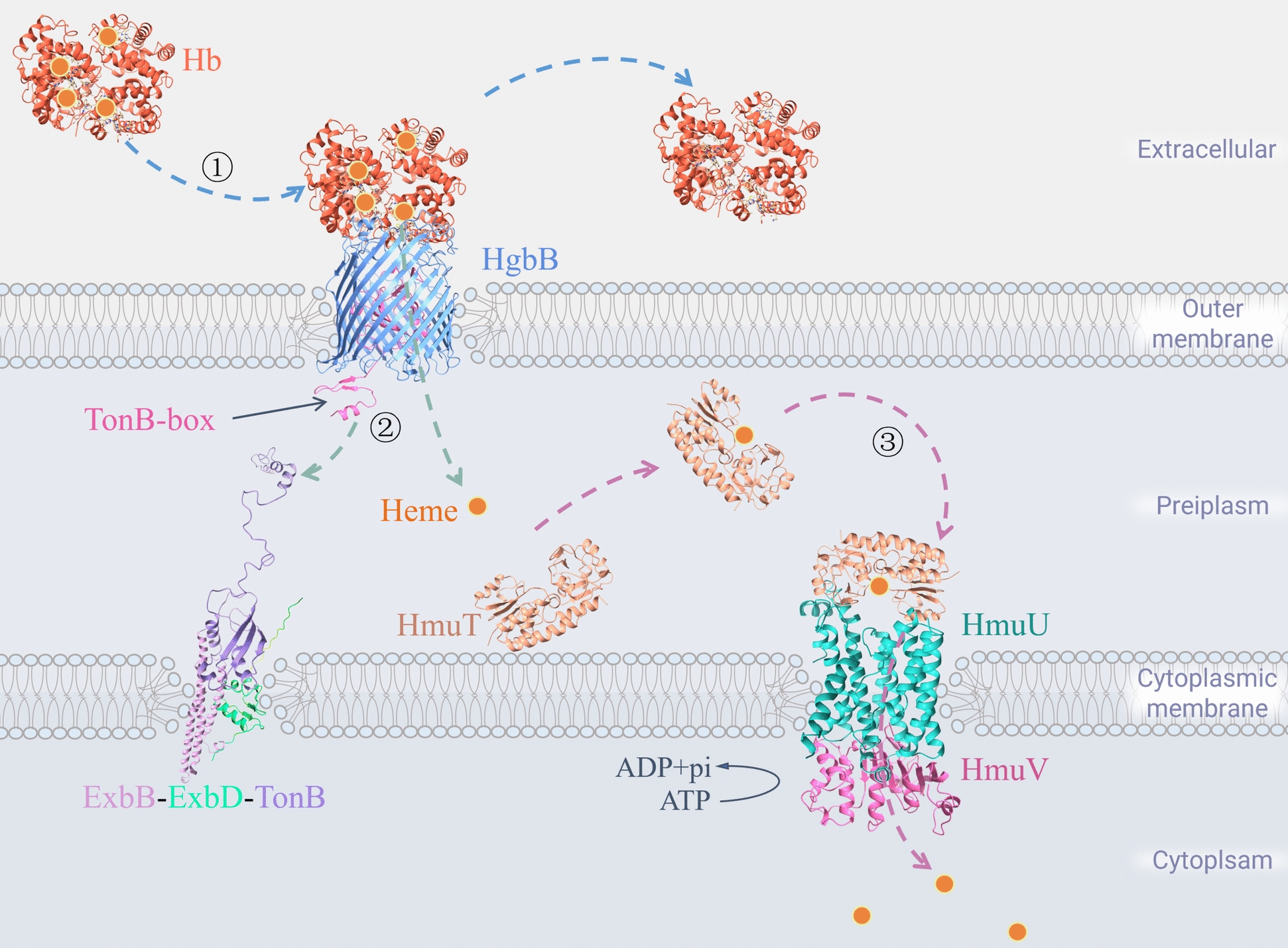
**Process of direct heme transport in** ***P. multocida*.**

Under iron-limiting conditions, *P. multocida* expresses Hb-binding protein A (HgbA), HgbB, Hb–hemopexin receptor, and other heme receptors [50]. These heme receptors are physically identical to TbpA and can directly recognize host Hb [51]. After the C-terminal β-barrel structural domain of the heme receptors binds to host Hb, the TonB-box region of the N-terminal plug structural domain undergoes a conformational change and extends into the periplasm of *P. multocida*. It binds to the C-terminal structural domain of TonB, which receives energy from the ExbB–ExbD–TonB complex, leads to opening of the plug structural domain of the heme receptors, and formation of a channel for heme transport [52] (Figure 2).

The transport of heme into the cytoplasm of *P. multocida* via the ABC transport system (HmuTUV) is poorly understood. However, the HmuTUV in *Yersinia pestis* has been largely elucidated. Heme is transported to the periplasm, where it is captured by the heme-binding protein HmuT and delivered to the transmembrane protein HmuU on the cytoplasmic membrane. While the ATP-binding protein HmuV hydrolyzes ATP to provide energy for HmuU, heme is transported to the cytoplasm [53, 54]. Heme catabolic enzymes break down heme as it enters the cytoplasm, thereby releasing Fe^2+^ for bacterial metabolism [55]. The GenBank-registered genomes of *P. multocida* (MAPT01000007.1, MANI01000004.1, MAPS01000002.1, etc.) all have homologous clusters of HmuTUV protein genes, whose annotated information predicts that they have the same ABC transporter system function.

### Indirect heme transport system

The indirect heme transport system, mediated by hemophores, is more efficient than the direct transport method. Hemophores are a type of protein released extracellularly by Gram-negative bacteria, including *Pseudomonas aeruginosa*, *Yersinia pestis*, and *Serratia marcescens*. The primary hemophores are HasA, HusA, HxuA, and HphA [56–60]. These proteins have a high affinity for heme and can compete for it in host Hb, myoglobin, and cytochrome before delivering it to specialized heme receptors on the bacterial outer membrane (different hemophores correspond to different receptors) [61]. The energy provided by the ExbB–ExbD–TonB complex to the heme receptor causes the plug structural domain to open and a channel to form, transporting heme to the periplasm and then to the cytoplasm for bacterial metabolism by the ABC transport system; a process that is identical to that of the direct heme transport system. The protein HasA, encoded by the gene *hasA*, is located in the Has system, which also contains the receptor for HasA, the heme acquisition system receptor (HasR). HasR acquires heme either free or via an extracellular heme transporter, the hemophore HasA [62]. Current research reports related to *P. multocida* indicate the presence of HasR in this bacterium, although that of HasA is uncertain [63]. Therefore, further studies are needed to determine whether an indirect heme transport mechanism occurs in *P. multocida*.

## Mechanism of iron uptake by iron carrier of *P. multocida*

Siderophores are high-affinity, low-molecular-weight metal chelators generated by microorganisms that typically bind to Fe^3+^ in the host or environment to create Fe^3+^-siderophore chelates, but they can also form chelates with other metal elements (such as molybdenum, manganese, cobalt, and nickel) [64, 65]. Siderophores can be categorized based on differences in chemical properties: (1) hydroxamates (consisting of acylated and hydroxylated alkylamines); (2) catecholates (consisting of catecholates and hydroxyls); and (3) carboxylates (consisting of citric acid or β-hydroxyaspartic acid). In addition to the categories listed above, some siderophores can be classified as mixed types, which are usually combinations of hydroxamates–catecholates or hydroxamates–carboxylates. The catecholate siderophore is the most effective at binding iron, whereas the carboxylate siderophore is the weakest [66].

**Figure 3:**
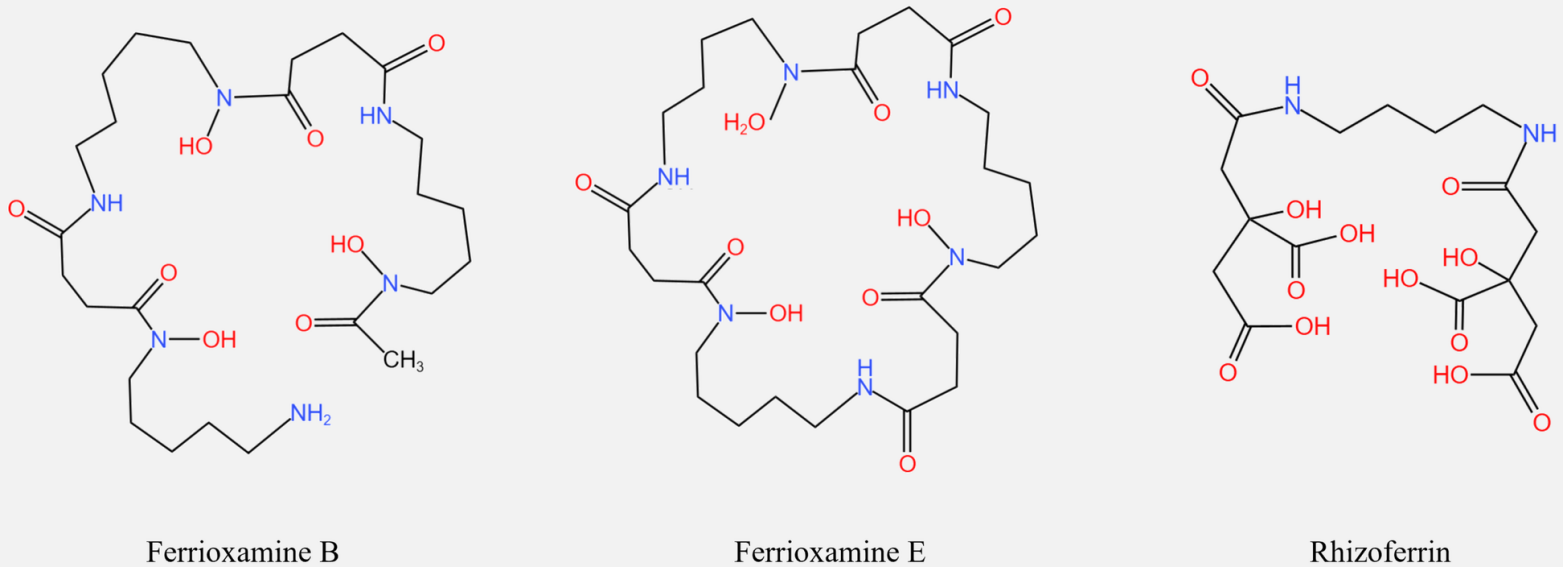
**Molecular structure of siderophores that can be used by *P. multocida.*** ferrioxamine B, a member of the hydroxamates siderophores; ferrioxamine E, a member of the hydroxamates siderophores; rhizoferrin, a member of the complex siderophores of hydroxamates-carboxylates.

Under iron-restricted conditions, *P. multocida* serotype A strains produce a carboxylate siderophore named multocidin [67]. Its receptor proteins are only identified as iron-associated outer membrane proteins that weigh 76, 84, and 94 kDa, and the detailed mechanism of the siderophore uptake is unknown [68]. *P. multocida* is unable to produce hydroxamate siderophores, but it can absorb ferrioxamine B and E. The strain does not produce catecholate siderophores, but it can utilize many metabolic intermediates, including dihydroxybenzoic acid from these siderophores. It is unclear if *P. multocida* produces mixed siderophores, despite the fact that they may absorb mixed siderophores such as rhizoferrin [69] (Figure 3).

FecA, a siderophore receptor, is found on the outer membrane of *P. multocida* [7]. In *E. coli*, FecA has a structure similar to that of the Tf receptor, TbpA, and the heme receptor, HgbA. It consists of a C-terminal β-barrel structural domain and an N-terminal plug structural domain [70]. FecA recognizes and binds siderophores, and after acquiring energy from the ExbB–ExbD–TonB complex, its plug domain opens, creating a pathway that transports ferric citrate to the periplasm and then to the cytoplasm via the ABC transport system of siderophore (FecBDE) [71, 72]. In the cytoplasm, Fe^3+^ is reduced to Fe^2+^ and separated from the siderophores for bacterial metabolism, while the siderophores are degraded or expelled [73, 74]. *P. multocida* carries the siderophore receptor FecA, which is similar to *E. coli*; hence, it is hypothesized that the FecA-mediated siderophore transport mechanism of *P. multocida* is similar to that of *E. coli*.

## Bacterial iron uptake regulator

Iron deficiency is detrimental to bacterial growth and reproduction, but excessive Fe^2+^ intake can trigger the Fenton reaction, which produces reactive oxygen species and reactive nitrogen, resulting in amino acid residue oxidation, protein and DNA damage, and, eventually, death [75]. As a result, the concentration of iron ions in bacteria must be strictly controlled. To maintain iron homeostasis, bacteria have evolved iron-related regulators such as the ferric uptake regulator (Fur) [76], iron-dependent regulator, iron response regulator [77], rhizobial iron regulator A [78], and iron–sulfur cluster regulator [79]. Currently, only Fur has been identified in *P. multocida*, which is the most important transcriptional regulator for maintaining iron homeostasis in bacteria. Fur has a dimerized metal ion-binding domain at the C terminus and a DNA-binding domain at the N terminus, and it represses or activates the transcription of iron-related genes in bacteria by sensing intracellular Fe^2+^ levels [80]. In the following section, we elaborate on Fur.

### Regulatory mechanisms of Fur as a transcriptional repressor

**Figure 4:**
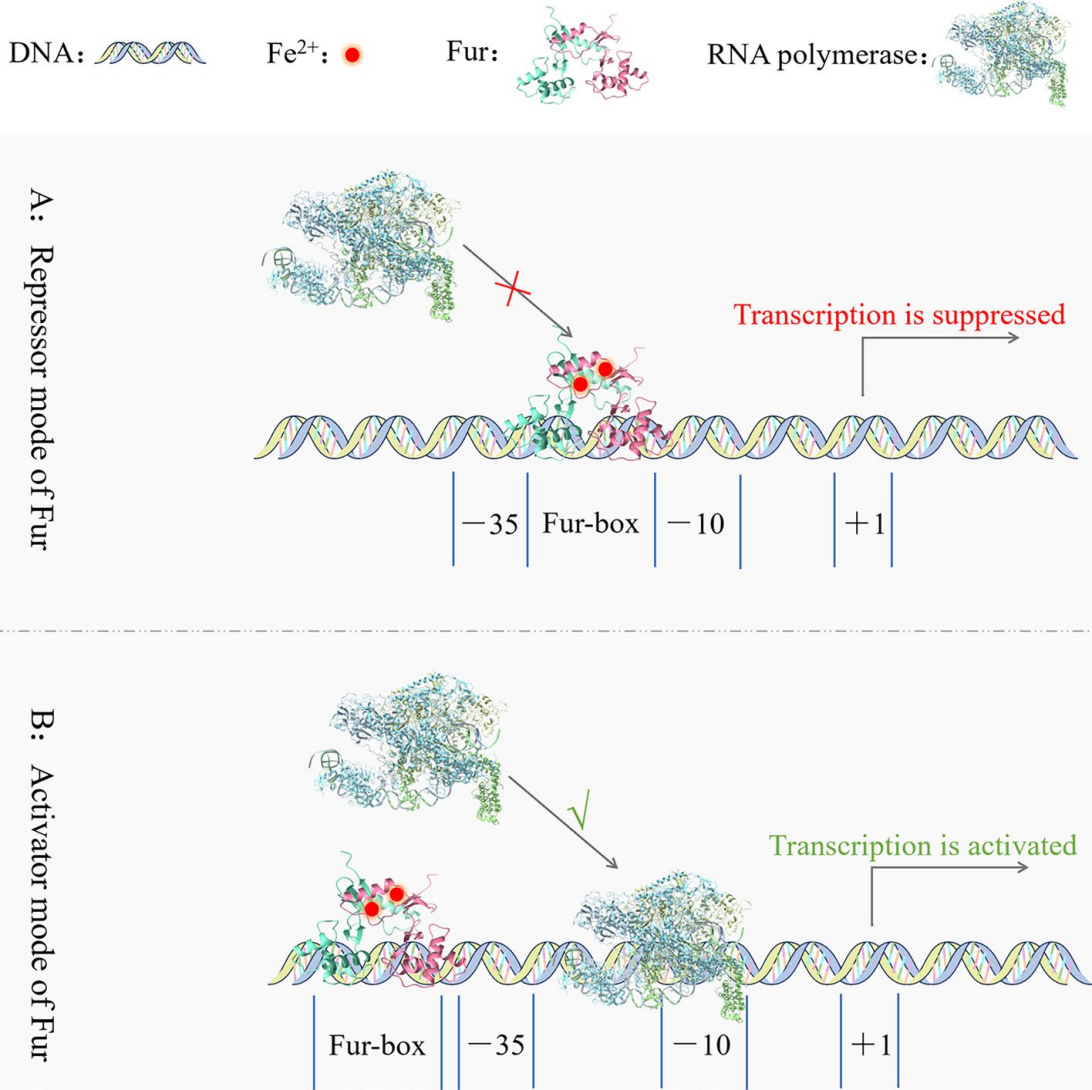
**Regulatory mechanism of Fur acts as transcriptional repressor (A) and transcriptional activator (B).**

When Fe^2+^ levels in bacteria are too high, Fur can detect and attach to a consensus sequence called the Fur-box on DNA. If the promoter on the DNA overlaps the site of the Fur-box, Fur occupies the promoter region, preventing RNA polymerase from binding to the DNA and inhibiting transcription [81, 82] (Figure 4A). In *E. coli*, for example, the Fur C terminus binds to Fe^2+^ to form a Fur–Fe^2+^ complex, prompting the DNA-binding domain at the N terminus of Fur to bind to the Fur-box on the *E. coli* siderophore-synthesis-associated gene cluster, *iucABCD*. This results in inability of RNA polymerase to aggregate to the promoter of *iucABCD*, inhibiting siderophore synthesis and preventing the bacterium from absorbing iron ions [83, 84]. Fur research in *P. multocida* has trailed behind, with exploration of the bacterial Fur beginning in 2001 [85]. Subsequently, researchers discovered the shared Fur-box sequence in the iron-related genes *tbpA*, *hgbA*, and *hgbB* of *P. multocida*; the expression of which is inhibited by Fur [86–88]. In addition, the synthesis of siderophores in *P. multocida* is also negatively regulated by Fur [89].

### Regulatory mechanisms of Fur as a transcriptional activator

When the Fur-box is positioned around −100 bp upstream of the transcription start point, Fur can enhance RNA polymerase aggregation at the −10 bp and −35 bp regions of DNA, hence activating DNA transcription [82, 90] (Figure 4B). Fur promotes the expression of the iron storage-related protein bacterioferritin (Bfr) [91]. Bfr can oxidize Fe^2+^ to Fe^3+^ and store it, preventing bacteria from being damaged by excess Fe^2+^ [92]. Bfr is classified into two types: heme-free ferritin A (FtnA) and heme-containing bacterioferritin B (BfrB) [93]. In *E. coli*, for example, Fur forms a Fur–Fe^2+^ complex with Fe^2+^, which binds to the Fur-box at −84 bp upstream of the bacterioferritin gene *ftnA* and activates *ftnA* transcription, thereby storing redundant free iron in the cytoplasm [94]. A homologous gene expressing FtnA also exists in *P. multocida*, and therefore [7], a transcriptional activation regulatory function of Fur may also exist in *P. multocida*.

## Summary and prospects

Pasteurellosis is an important bacterial infectious disease that affects a wide range of animals. In this paper, three iron uptake mechanisms of *P. multocida* are described in detail. This provides a systematic theoretical basis for a comprehensive understanding of the molecular pathogenesis of the iron uptake systems of *P. multocida*, as well as new ideas for the development molecular-targeted drugs and subunit vaccines against *P. multocida*. After analyzing research on the iron uptake systems of *P. multocida* and its occurrence and regulatory mechanism, we identified the following issues that require immediate attention. (1) Many bacteria rely on siderophores for iron uptake, and research into their chemical structures has advanced significantly in *E. coli* and *Rhizobium*. However, the research on multocidin, the siderophore of *P. multocida*, is still ambiguous, and there is a significant knowledge gap in the understanding of its chemical structure, synthetic and secretory pathway, and outer membrane receptor; all of which require urgent further study and clarification. At the same time, it is necessary to strengthen the investigation and development of *P. multocida* siderophore production pathways to produce a diverse range of novel siderophores. (2) The transport efficiency of the heme indirect transport system mediated by the hemophores is higher than that of the heme direct transport system. However, there is a lack of research on the hemophores of *P. multocida*, and the detailed mechanism of the hemophores mediating the uptake of heme from the host heme binding protein requires further investigation. (3) The distribution of iron-associated outer membrane receptors such as TbpA, HgbA, and HgbB in different regions, hosts, and serotypes of *P. multocida* strains differs significantly [95–97]. To prevent and control pasteurellosis, the molecular epidemiology of *P. multocida* must be thoroughly investigated and analyzed. (4) Although Fur has been extensively investigated in many bacteria and more Fur-regulated genes have been identified, there are still many molecular mechanisms and strategies of regulation that have not been fully elucidated [89]. The effect on virulence after the deletion of *fur* gene in *P. multocida* is significantly less pronounced than that of other bacteria, implying that this bacterium may have other regulators that need to be investigated.

The current important directions in the application of the bacterial iron uptake mechanisms include: (1) developing new drug targets by inhibiting siderophore synthesis or blocking the iron uptake pathway of pathogenic bacteria; (2) utilizing synthetic siderophore–drug conjugates to aid drug translocation into pathogenic bacterial cells to improve the treatment of multidrug-resistant bacterial infections; (3) designing subunit vaccines with single or multiple antigenic components, or synthetic peptide vaccines (multi-epitope vaccines); and (4) exploiting iron-related genes as a breakthrough to build novel attenuated vaccines with gene deletion. Understanding role and regulatory mechanism of *P. multocida* in acquiring nutrients such as iron from the host will help with the prevention and control of pasteurellosis.

## Abbreviations

Tf: transferrin
Hb: hemoglobin
RirA: rhizobial iron regulator A
TbpA: transferrin-binding protein A
holo-Tf: iron-containing Transferrin
ABC transporter: ATP-binding cassette (ABC) transporter
FbpA: ferric binding protein A
HgbA: hemoglobin-binding protein A
HgbB: hemoglobin-binding protein B
HemR: hemoglobin–hemopexin receptor
HasR: heme acquisition system receptor
Fur: ferric uptake regulator
FtnA: heme-free ferritin A
BfrB: heme-containing bacterioferritin B
